# Doing the Work—or Not: The Promise and Limitations of Diversity, Equity, and Inclusion in US Medical Schools and Academic Medical Centers

**DOI:** 10.3389/fpubh.2022.900283

**Published:** 2022-06-22

**Authors:** Caitlin Jade Esparza, Mark Simon, Eraka Bath, Michelle Ko

**Affiliations:** ^1^School of Medicine, University of California, Davis, Sacramento, CA, United States; ^2^Storywalkers Consulting, Davis, CA, United States; ^3^The Jane and Terry Semel Institute for Neuroscience and Human Behavior at UCLA, Department of Psychiatry, David Geffen School of Medicine at University of California, Los Angeles, Los Angeles, CA, United States; ^4^Division of Health Policy and Management, Department of Public Health Sciences, University of California, Davis, Davis, CA, United States

**Keywords:** diversity, equity, inclusion, medical schools, academic medicine

## Abstract

While the number of positions, committees, and projects described as “Diversity, Equity, and Inclusion (DEI)” work has grown rapidly in recent years, there has been little attention to the theory, praxis, or lived experience of this work. In this perspective, we briefly summarize the research and concepts put forth by DEI leaders in higher education more broadly, followed by an analysis of the literature's application to academic medicine. We then discuss the ways in which language obscures the nature of DEI and the necessity of scholarship to evaluate the extensive range of practices, policies, statements, and programs the label is given to.

## Introduction

The words “diversity, equity, and inclusion” (DEI) are frequently linked together in academic medicine to encompass everything from admission guidelines, to curriculum, to mistreatment policies. Yet despite years of solidarity statements and highly publicized promises, academic medicine has only recently begun to visibly pursue “Diversity, Equity, and Inclusion.” For decades, the DEI work of scholars, community members, and trainees was rarely acknowledged, much less adequately resourced or compensated-even when institutions' progress has been reliant on their labor. Even formal DEI positions often lack clear expectations and the responsibilities associated with these roles have largely not been defined or codified, particularly beyond the executive level (1). Additionally, there is a noticeable lack of scholarship to interrogate the nature of these efforts, potential foundational frameworks and best practices, or meaningful outcomes to measure progress.

### The DEI Landscape Today

To better understand emerging trends, between December 2020 and September 2021 we interviewed DEI leaders from medical schools and academic medical centers across the US on their experiences, motivations, and available resources. Interviewees shared that DEI work in academic medicine currently has a wide breadth of definitions and manifestations, with individuals and teams working in almost every possible institutional space, and with dramatic variability in level of authority, financial support, dedicated staffing, and scope. Directives from their leadership tended toward broad generalities, such as “create a culture of inclusion” and “create an environment where everybody feels welcome and included” rather than specific policies or measurable outcomes (2).

The experiences shared by DEI leaders in academic medicine are consistent with the work of scholars in post-secondary education and industry. Prior research has pointed to the lack of coordinated effort, clear objectives, sufficient funding, and adequate staffing as limitations to the effectiveness of diversity initiatives (3). Others have described how institutions dilute the meaning of diversity from the field's origins in affirmative action, creating instead a collection of words that can mean both everything and nothing at all (4). Leaders fail to consider how Black, Latinx, and Indigenous trainees, faculty, and staff are harmed by institutions that were built, and are maintained, with the tools of white supremacy, settler colonialism, capitalism, and patriarchy. Sociologist James Thomas explains how institutions create “diversity regimes” that link “diversity” to institutional identity without the tangible commitments to functionally do so (5). As a result, diversity devolves to re-branding, rather than a core value or set of practices.

Common DEI efforts, such as establishing new administrative positions, departmental committees, or implementation of health equity curricula, are not inherently flawed approaches (6–17). However, when institutions adopt initiatives in the absence of evidence-based strategic planning or structural reinforcement, their potential impact is severely limited. In addition, the work typically falls on a single or small group of minoritized trainees, faculty, and staff (18–21). The placement of responsibility on a select few—rather than the entirety of institutional, departmental, and programmatic leadership—is neither sustainable nor equitable. This clear manifestation of minority tax, alongside the daily abuse and discrimination experienced by minoritized people in academia, leads to burnout and exit (22–30). In turn, an institution's entire DEI programming may stall or completely halt with the loss of only a handful of individuals.

Institutional accountability largely relies on diversity-related accreditation standards such as those established by the Liaison Committee on Medical Education (LCME) and Accreditation Council for Graduate Medical Education (ACGME) despite their vague, non-metric driven construction and unclear effectiveness (2, 31–34).

It is essential that the burden of responsibility and accountability to DEI work lay with the institution and its leaders. Identifying people with expertise and lived experience to consult or lead DEI initiatives is crucial, but not sufficient. Below we describe how the unexamined, vague language of “DEI” is inherently limiting to the design and implementation of DEI efforts.

### Language Matters… to a Point

The language we choose can bolster or undermine our work. The phrase “diversity, equity, and inclusion” is made up of three distinct concepts, yet they are usually lumped together to reference a range of ideas and practices with no functional definition.

In some contexts, this ambiguity has allowed for the weaponization of language, such as the excusal of racist rhetoric under the guise of a “diversity of opinions” (3). This lack of specificity also further weakens productive dialogue, allowing for an incident of discrimination or abuse to be treated as an isolated “disagreement” or event and relegated to the DEI office to manage, or ignored completely (18).

Furthermore, each concept within DEI does not receive equal emphasis. The concept of diversity garners the lion's share of attention in academic medicine, vs. “equity” or “inclusion”—and in recent years “anti-racism” and “justice” (35). While there is value in expanding our vocabulary—particularly when seeking to understand and challenge complex systems of oppression—doing so does not inherently lead to action. An inconsistent understanding and application of language not only hinders our ability to communicate but fosters new opportunities for manipulation, obfuscation, and avoidance of responsibility by institutions and their leadership.

In the months following the murders of George Floyd, Breonna Taylor, and Ahmaud Arbery in 2020, many academic medicine institutions released statements in response to public demand. However, in an analysis of 45 such statements, researchers found that one third did not include the words “racism” or “racist.” Of those that did, many minimized institutional accountability by focusing on interpersonal racism, and offered no commitments to specific actions. In addition, many used the words “diversity, equity, and inclusion,” or highlighted past DEI work unrelated to racial justice, rather than explicitly address anti-Black racism (36). In other words, following a clear national moment of racial reckoning, academic medical institutions were unable or unwilling to name racism, failed to acknowledge medicine's role in perpetuating racial violence and structural inequities, and instead often conflated DEI work with anti-racism.

A specific example of misalignment between an institution's stated values and observed action can be seen in the case of Dr. Aysha Khoury, a Black faculty member who was suspended and ultimately dismissed by Kaiser Permanente Bernard J. Tyson School of Medicine (KPSOM) in August 2020 after sharing institutional and personal experiences of racism in a small group discussion. Dr. Khoury was asked to facilitate the session and include content regarding the “legacies of power structures and institutionalized racism that result in gender bias and race bias in medicine today” (37). However, within hours of the session Dr. Khoury was notified that she was to be suspended from teaching and clinical duties due to a complaint regarding the discussion. Dr. Khoury had joined the KPSOM as an inaugural faculty member in 2019 and just 10 weeks prior to her suspension had been notified that she would be considered for an increase in academic rank. Despite student and community support—including all eight students from the discussion group—Dr. Khoury was denied appointment renewal in December 2020 and her case for wrongful termination against KPSOM is ongoing (37–39). As of Spring 2022, the KPSOM website includes among its values “promoting inclusiveness and diversity in medical education and the health professions… [and] advocating for change in medical education and the health professions” (40).

Academic medicine leaders may avoid acknowledging institutional responsibility because this would associate them with the forces that create, uphold, and perpetuate inequitable systems—namely white supremacy, misogyny, ableism, classism, and homophobia (41, 42). However, these are the same forces that wrote medicine's violent history and continue to uphold inequitable healthcare systems. The catch-all term, “diversity, equity and inclusion” can thus allow institutions to hide behind language and skirt the difficult work of examining and uprooting the foundations upon which medicine has accumulated and concentrated power (43–48).

### A Closer Look

Unexamined language is reflected in unexamined work. For example, an institution could create a new DEI office, provide funding, and issue a directive to bring the percentage of students “underrepresented in medicine” in their next matriculating class to a level that parallels the state's demographics (49). However, if the push to reach this representation goal is not matched by investment in the learning environment—i.e., diversity without inclusion or equity—the harm to those students would be significant.

Multiple studies have found that trainees minoritized by their race, gender, or sexuality experience severe career and psychosocial consequences in academic medicine, including mistreatment, discrimination in evaluations, and high rates of depressive and anxiety symptoms. Students navigate rigid, ableist expectations of medical training without adequate support or culturally responsive resources (50–57). This highlights an intersectional challenge—that is one which is formed by overlapping axes of oppression—and therefore requires an appropriately robust and adaptable set of solutions.

For example, a program can be designed to provide trainees access to therapy, including to providers who reflect the identities and experiences of trainees. However, such an investment must also be paired with structural changes, such as protected time, to allow for actual access and benefit. Program and academic leave policies that limit excusals, require extensive documentation, or do not explicitly include mental health care as equivalent in validity to other areas of medical treatment are inherently ableist. So, while hiring providers who share the marginalized identities of trainees might partially mitigate inequitable access to culturally responsive care, it does not adequately consider structural design that can multiply the barriers that minoritized trainees, in particular disabled and chronically ill trainees, experience while in medical training.

Institutions must invest time and resources to remove, redesign, and replace the systems that have been established to center and prioritize the needs of predominantly white able-bodied cisgender men. Otherwise, increased representation merely magnifies the same cycles of abuse, marginalization, and harm. Diversity alone cannot fulfill the promise of equity or inclusion.

### Looking Inward While Moving Forward

Academic medicine needs to use the rigor of scholarship to understand what diversity, equity, inclusion, justice, anti-racism, and other such concepts mean and how they can be actualized. Such scholarship is critical to develop frameworks and models to move institutions beyond performative DEI policies, mission and solidarity statements, and toward actual theory-grounded praxis and sustainable, transformative organizational change.

Academic medicine must look beyond its own expertise and collaborate with scholars of other fields, particularly the social sciences. Academic medicine has a self-defeating tendency to treat non-clinical research as minimally relevant (58–60). However, academic medicine, like other institutional structures, is a social construction. Analysis of institutional power dynamics remains woefully absent, despite its centrality to other fields such as critical race and feminist theory (42, 61–63). To understand how academic medicine has institutionalized racism, reinforced gender biases, constructed barriers to accessibility, and so much more, we need to recognize and seek the help of experts in those very issues.

Finally, we must consider the intention and value of the questions we choose to ask. As a start, we need to define the field—what does DEI mean, what are trying to achieve, and why? We still lack a clear picture of what DEI in academic medicine currently encompasses—what are we doing and how are we doing it? With these pieces of information, we can begin to assess the ways in which the work- DEI or not- aligns with stated objectives. We can then move on to the systematic and rigorous evaluation of specific programs and policies for their impacts. There are many ways to move forward, and an abundance of unanswered questions, but these overarching inquiries will allow us to create a foundation upon which to rebuild.

## Discussion

Meaningful progress in academic medicine will require stepping away from *ad hoc* DEI programs and moving toward work grounded in theories of change, supported by evidence, and constantly interrogated for purpose, operationalization, and impact.

While we push for investment in the rigorous analysis of DEI work in academic medicine, that must not come at the expense of progress. When there is so much left undone and uninterrogated, there is a risk of limiting action for the sake of reflection. Given the ongoing harm experienced by those currently working and training in our field, we can not allow such a delay and must balance careful examination with continued action.

The reality of the current academic medicine landscape is one that was designed by and for cis-gender, heterosexual, non-disabled white men. The effects of such a history are baked into our current reality and while the manifestation of such a construction differs significantly depending on who or what the subject is, across marginalized identities and experiences there is abuse, discrimination, and exclusion. We keep out poor people with ever increasing tuition costs and debt burdens, we withhold power from Black people by limiting leadership representation to designated DEI roles, we hinder disabled students' learning by gatekeeping accommodations and failing to offer remote learning options, and we suppress the expression of our queer colleagues with racist and gendered professionalism standards (51, 64–66). The active exclusion, discrimination, and harm created by the medical education system are even more complexly entrenched when considered through a lens of intersectionality, revealing a built environment that is near impossible to enter and incredibly traumatic for those with multiple marginalized identities. Until we approach DEI in academic medicine with the rigor, intentionality, and investment needed to evaluate and influence an exceedingly complex and adaptable ecosystem of oppression, meaningful change will continue to elude us.

## Data Availability Statement

The original contributions presented in the study are included in the article/supplementary material, further inquiries can be directed to the corresponding author/s.

## Author Contributions

MS: research team member, conceptual development, and draft review. EB and MK: research team advisor/co-PI, conceptual development, and draft review. All authors contributed to the article and approved the submitted version.

## Funding

This work was supported by the Health Resources and Services Administration (HRSA) of the U.S. Department of Health and Human Services (HHS) as part of an award totaling $3,791,026 with 0% financed with non-governmental sources. Grant ID UH1HP29965. The contents are those of the author(s) and do not necessarily represent the official views of, nor an endorsement, by HRSA, HHS, or the U.S. government. For more information, please visit HRSA.gov. This work was also supported by the University of California, Davis Office for Health Equity, Diversity, and Inclusion (HEDI) and University of California, Davis Office for Diversity, Equity, and Inclusion.

## Conflict of Interest

MS was employed by Storywalkers Consulting. The remaining authors declare that the research was conducted in the absence of any commercial or financial relationships that could be construed as a potential conflict of interest.

## Publisher's Note

All claims expressed in this article are solely those of the authors and do not necessarily represent those of their affiliated organizations, or those of the publisher, the editors and the reviewers. Any product that may be evaluated in this article, or claim that may be made by its manufacturer, is not guaranteed or endorsed by the publisher.
